# Inhibition of mPGES-1 attenuates efficient resolution of acute inflammation by enhancing CX3CL1 expression

**DOI:** 10.1038/s41419-021-03423-2

**Published:** 2021-02-02

**Authors:** Peter Rappl, Silvia Rösser, Patrick Maul, Rebekka Bauer, Arnaud Huard, Yannick Schreiber, Dominique Thomas, Gerd Geisslinger, Per-Johan Jakobsson, Andreas Weigert, Bernhard Brüne, Tobias Schmid

**Affiliations:** 1 Institute of Biochemistry I, Faculty of Medicine, Goethe-University Frankfurt, Frankfurt, Germany; 2 Fraunhofer Institute for Translational Medicine and Pharmacology, Frankfurt, Germany; 3 Institute of Clinical Pharmacology, pharmazentrum Frankfurt/ZAFES, University Hospital, Goethe-University Frankfurt, Frankfurt, Germany; 4 Rheumatology Unit, Dep. of Medicine, Solna, Karolinska Institutet, Karolinska University Hospital, Stockholm, Sweden; 5 German Cancer Consortium (DKTK), Partner Site Frankfurt, Frankfurt, Germany; 6 Frankfurt Cancer Institute, Goethe-University Frankfurt, Frankfurt, Germany

**Keywords:** Acute inflammation, Peritoneal macrophages

## Abstract

Despite the progress to understand inflammatory reactions, mechanisms causing their resolution remain poorly understood. Prostanoids, especially prostaglandin E_2_ (PGE_2_), are well-characterized mediators of inflammation. PGE_2_ is produced in an inducible manner in macrophages (Mϕ) by microsomal PGE_2_-synthase-1 (mPGES-1), with the notion that it also conveys pro-resolving properties. We aimed to characterize the role of mPGES-1 during resolution of acute, zymosan-induced peritonitis. Experimentally, we applied the mPGES-1 inhibitor compound III (CIII) once the inflammatory response was established and confirmed its potent PGE_2_-blocking efficacy. mPGES-1 inhibition resulted in an incomplete removal of neutrophils and a concomitant increase in monocytes and Mϕ during the resolution process. The mRNA-seq analysis identified enhanced C-X3-C motif receptor 1 (CX3CR1) expression in resident and infiltrating Mϕ upon mPGES-1 inhibition. Besides elevated *Cx3cr1* expression, its ligand CX3CL1 was enriched in the peritoneal lavage of the mice, produced by epithelial cells upon mPGES-1 inhibition. CX3CL1 not only increased adhesion and survival of Mϕ but its neutralization also completely reversed elevated inflammatory cell numbers, thereby normalizing the cellular, peritoneal composition during resolution. Our data suggest that mPGES-1-derived PGE_2_ contributes to the resolution of inflammation by preventing CX3CL1-mediated retention of activated myeloid cells at sites of injury.

## Introduction

Inflammatory responses are essential for an effective host defense against pathogenic threats such as invading bacteria or viruses^1,2^. The onset of acute inflammation is typically characterized by a rapid and fulminant influx of neutrophils, i.e. polymorphonuclear cells (PMN), into inflamed tissues, where they form a first line of defense to eliminate pathogens. Thereafter, monocytes (MO) are recruited, followed by their differentiation into distinct macrophage (Mϕ) subsets. Mϕ not only phagocytose invading pathogens and present them via their major histocompatibility complexes (MHC) to the adaptive immune system, they also remove short-lived, dying neutrophils in a process known as efferocytosis^3–5^. The uptake of apoptotic cells alters the phenotype of Mϕ from a pro-inflammatory to an immune-regulatory one^6–9^, thereby contributing to the resolution of inflammation, i.e. normalization of tissue homeostasis^10–12^. Effective resolution of inflammation is important to avoid tissue damage by producing uncontrolled destructive signals. Unfortunately, the transition from inflammation to its resolution remains incompletely understood. There is increasing evidence that the resolution phase is already initiated early during onset of inflammation and that interfering with early inflammatory reactions alters resolution as well^11,13^. Along these lines, recent studies indicated that the well-established pro-inflammatory lipid mediator prostaglandin E_2_ (PGE_2_)^14^ also bears pro-resolving properties^15–20^. This is of interest, since non-steroidal anti-inflammatory drugs (NSAIDs), which are the main class of non-prescribed, over the counter anti-inflammatory drugs, target cyclooxygenases to inhibit the production of prostanoids, most notably PGE_2_^21,22^. More specific inhibitors of PGE_2_ production have recently been developed to target the terminal synthase active under inflammatory conditions, i.e. the microsomal PGE_2_ synthase-1 (mPGES-1)^23–25^.

In this study, we explored the role of PGE_2_ during resolution of inflammation. Experimentally, we blocked PGE_2_ production in a zymosan-induced peritonitis model, selectively during the resolution phase using a specific mPGES-1 inhibitor^26^.

## Results

### Inhibition of mPGES-1 alters resolution of zymosan-induced peritonitis

**Fig. 1:**
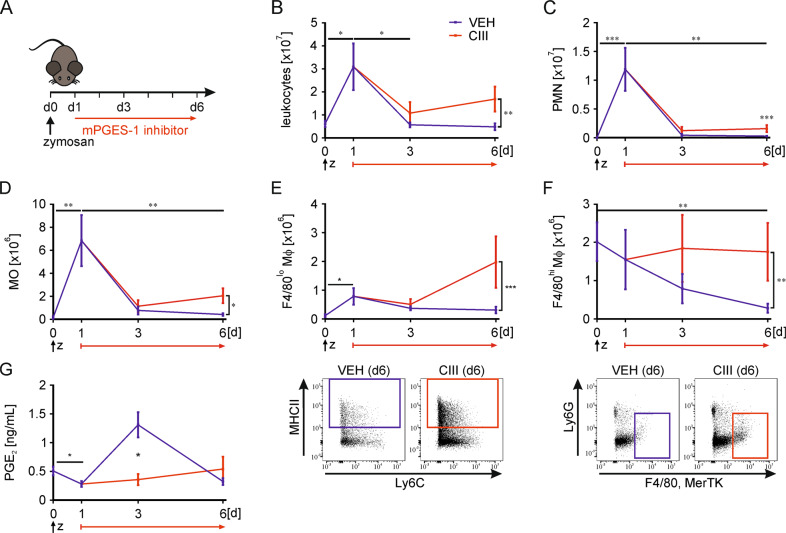
Inhibition of mPGES-1 alters the cellular composition during resolution of zymosan-induced peritonitis. **A** Starting 24 h post i.p. zymosan (5 mg/kg) injection, mice received daily i.p. injections of the mPGES-1 inhibitor compound III (CIII) (25 mg/kg) or the appropriate vehicle control (VEH). **B–F** Cellular composition of the peritoneal lavages was analyzed by FACS. Numbers of viable (**B**) leukocytes (CD45^+^), (**C**) PMNs (CD11b^+^, Ly6G^+^), (**D**) monocytes (MO; Ly6G^lo^, F4/80^lo^, MerTK^lo^, MHCII^–^), (**E**) F4/80^lo^ (Ly6G^lo^, F4/80^lo^, MerTK^lo^), and (**F**) F4/80^hi^ (Ly6G^lo^, F4/80^hi^, MerTK^hi^) macrophages (Mϕ) in the peritoneum. Representative dot-blots of (**E**, *lower panel*) F4/80^lo^ Mϕ and (**F**, *lower panel*) F4/80^hi^ Mϕ at day 6. **G** PGE_2_ concentrations in the peritoneal lavages were determined by LC/MS/MS. Data are represented as means ± SEM (*n* ≥ 6 per group). For statistical analysis within each treatment, one-way ANOVA with Holm–Sidak posthoc test was used for parametric data, otherwise, Kruskal–Wallis test was used. Analysis between the treatments for parametric data was done via two-way ANOVA with Holm–Sidak posthoc test, non-parametric data were log-transformed (*p < 0.05, **p < 0.01, ***p < 0.01).

While the role of prostanoids, especially PGE_2_, during the initial phase of inflammation is well characterized, its impact towards resolution of inflammation remains largely elusive. To better understand how PGE_2_ contributes to late inflammatory processes, we induced a self-limiting peritonitis in C57/BL6 mice by intraperitoneal (i.p.) injection of zymosan (5 mg/kg), and inhibited PGE_2_ synthesis by daily i.p. injections of the selective mPGES-1 inhibitor CIII (25 mg/kg)^26^ once the inflammatory response was established for 24 h (Fig. 1A). FACS analyses (gating strategy see Supplementary Fig. 1) supported the transient character of the peritonitis model as the massive increase of leukocytes (CD45^+^) observed at day 1 was reversed starting at day 3. Inhibition of mPGES-1 attenuated this decrease, resulting in higher leukocyte numbers in the peritoneum at days 3 and 6 (Fig. 1B). With respect to neutrophils (PMN), the major cellular infiltrates during inflammation, we noticed only a small increase in cell numbers comparing vehicle control (VEH)- vs. CIII-treated mice at day 6 (Fig. 1C). Since Mϕ coordinate early as well as late inflammatory processes, we next determined the presence of MO and Mϕ. There was a massive infiltration of MO at day 1, which returned to basal levels at day 6 in VEH-treated mice. In contrast, MO numbers started to increase again at day 6 compared to day 3 upon mPGES-1 inhibition (Fig. 1D). Importantly, two subsets of Mϕ are found in the peritoneum, F4/80^lo^, MerTK^lo^ Mϕ, which are considered as infiltrating Mϕ of monocytic origin, and F4/80^hi^, MerTK^hi^ Mϕ, which represent the resident Mϕ population, either of mesodermal descent or differentiated from infiltrating Mϕ^27,28^. In control animals, F4/80^lo^ Mϕ number behaved similarly to MO although their increase was less pronounced at day 1, returning to baseline levels at day 6 (Fig. 1E). CIII-treatment showed higher numbers of F4/80^lo^ Mϕ at day 3, which further increased at day 6 compared to controls. In contrast, the number of F4/80^hi^ Mϕ steadily decreased over time in control animals but remained at high, starting levels, when mPGES-1 was blocked (Fig. 1F). Determination of PGE_2_ concentrations in the peritoneal lavage validated the efficacy of CIII to prevent an increase of PGE_2_ especially at early times of resolution, i.e. at day 3 (Fig. 1G). It appears that PGE_2_ is needed to normalize the myeloid cell composition during resolution of inflammation in zymosan-induced, acute peritonitis.

### Inhibition of mPGES-1 alters the macrophage resolution phenotype

**Fig. 2:**
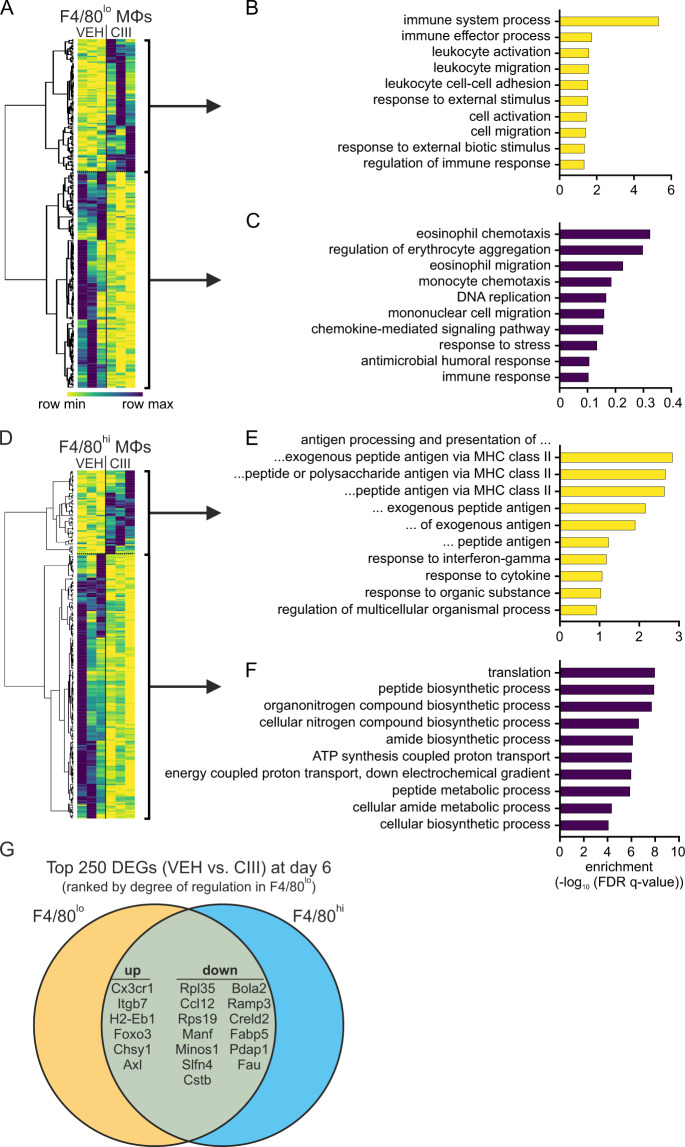
Peritoneal macrophages are activated during resolution of inflammation upon mPGES-1 inhibition. Starting 24 h post i.p. zymosan (5 mg/kg) injection, mice received daily i.p. injections of the mPGES-1 inhibitor compound III (CIII) (25 mg/kg) or the appropriate vehicle control (VEH). F4/80^hi^ and F4/80^lo^ macrophages (Mϕ) were FACS sorted followed by mRNA-seq analysis (*n* = 3). **A**, **D** mRNA expression of the top 250 differentially regulated genes comparing VEH- and CIII-treated mice at day 6 in **A** F4/80^lo^ and **D** F4/80^hi^ Mϕ. Top ten enriched GO terms based on the hierarchical clusters of upregulated (**B**) or downregulated **C** DEGs from F4/80^lo^ Mϕ. Top ten enriched GO terms based on the hierarchical clusters of upregulated (**E**) or downregulated **F** DEGs from F4/80^hi^ Mϕ. **G** Venn diagram of the top 250 DEGs in F4/80^lo^ (orange) and F4/80^hi^ Mϕ (blue). Overlapping DEGs (green) are ranked according to their degree of regulation in F4/80^lo^ Mϕ.

As PGE_2_ affects the number of Mϕ during resolution of inflammation, we were interested to explore whether their polarization is affected as well. To this end, we determined transcriptome changes in F4/80^lo^ and F4/80^hi^ Mϕ subpopulations (Fig. 1E, F) by mRNA-sequencing. Amongst the top 250 differentially expressed genes (DEGs) in F4/80^lo^ Mϕ (Fig. 2A, Supplementary Table 1) 95 genes were upregulated when mPGES-1 was blocked. These showed an enrichment in GO terms towards immune cell activation processes (Fig. 2B), while the downregulated genes revealed GO terms associated with chemotaxis and migration (Fig. 2C). In F4/80^hi^ Mϕ, the 60 upregulated candidates amongst the top 250 DEGs (Fig. 2D, Supplementary Table 2) showed a pronounced enrichment in processes linked to antigen processing and presentation via MHCII (Fig. 2E), while GO terms linked to metabolic processes were found in the downregulated targets (Fig. 2F). Interestingly, only 19 of the top 250 DEGs were shared between F4/80^lo^ and F4/80^hi^ Mϕ (Fig. 2G). Of these, six were upregulated (including CX3C motif chemokine receptor 1 (Cx3cr1), integrin β7 (Itgb7), and H2-Eb1), while 13 were downregulated upon mPGES-1 inhibition (including C–C motif chemokine ligand 12 (Ccl12) and fatty acid-binding protein 5 (Fabp5)).

These data imply that inhibition of mPGES-1 activity during resolution of inflammation not only affects inflammatory cell numbers but also their activation profile.

### Inhibition of mPGES-1 enhances the CX3CL1-CX3CR1 axis

**Fig. 3:**
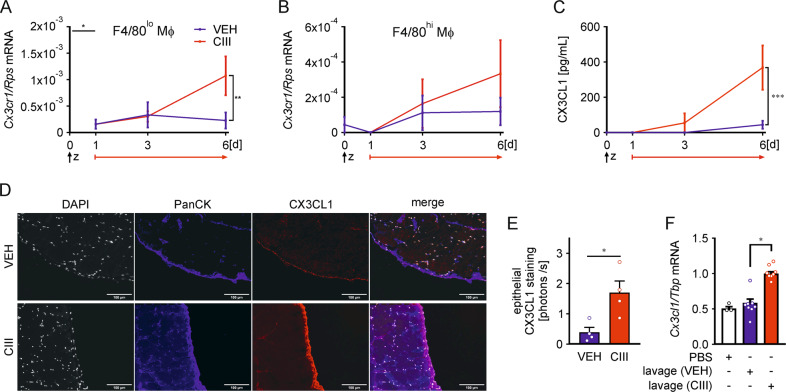
Inhibition of mPGES-1 enhances expression of the CX3CR1/CX3CL1 axis during resolution of inflammation. Starting 24 h post i.p. zymosan (5 mg/kg) injection, mice received daily i.p. injections of the mPGES-1 inhibitor compound III (CIII) (25 mg/kg) or the appropriate vehicle control (VEH). *Cx3cr1* mRNA expression in **A** F4/80^lo^ and **B** F4/80^hi^ macrophages (Mϕ) was analyzed by RT-qPCR analysis. **C** Concentrations of CX3CL1 in the peritoneal lavage were determined by ELISA. **D** Representative pictures of peritoneal membranes from VEH- and CIII-treated animals at day 6 using multiplexed IHC staining for the epithelial marker pan-cytokeratin (PanCK), CX3CL1, and DAPI. **E** Quantification of CX3CL1 staining intensity in PanCK-expressing epithelial cells using the InForm-software. **F** Epithelial E0771 cells were treated with medium supplemented with peritoneal lavages obtained from VEH- or CIII-treated mice at day 6 of the peritonitis model for 4 h. *Cx3cl1* mRNA expression was analyzed by RT-qPCR. Data are represented as means ± SEM (*n* ≥ 4 per group). For statistical analysis within each treatment, t test or one-way ANOVA with Holm–Sidak posthoc test was used for parametric data, otherwise, Kruskal–Wallis test was used. Analysis between the treatments for parametric data was done via two-way ANOVA with Holm–Sidak posthoc test, non-parametric data were log-transformed (*p < 0.05, **p < 0.01, ***p < 0.001).

Knowing that inhibition of mPGES-1 increases Mϕ numbers during resolution of inflammation, we next asked if the enhanced expression of the well-established myeloid chemokine receptor CX3CR1 might provide a mechanistic explanation. Validation of *Cx3cr1* mRNA kinetics supported a significant increase at day 6 upon CIII-treatment in FACS-sorted F4/80^lo^ (Fig. 3A) and F4/80^hi^ Mϕ (Fig. 3B), although less pronounced in the latter ones. Considering that there is only one known ligand for CX3CR1, namely CX3C motif chemokine ligand 1 (CX3CL1, also known as fractalkine)^29,30^, we determined the concentration of this chemokine in peritoneal lavages. CX3CL1 was absent in naive mice or 1 day after zymosan injection, remained low in VEH-treated animals, but continuously increased in mice that received CIII (Fig. 3C). Thus, limiting mPGES-1-derived PGE_2_ might attenuate the recruitment of Mϕ by interfering with the production of CX3CL1.

Since *Cx3cl1* mRNA was not detectable in any of the Mϕ subpopulations tested in our model, but has been shown as a product of epithelial and endothelial cells^31^, we isolated peritoneal membranes from mice at day 6 following zymosan injection and used multiplexed immunohistochemistry (IHC) to determine the local source of this chemokine. Co-localization of CX3CL1 with the epithelial marker pan-cytokeratin (PanCK) in both, VEH- and CIII-treated conditions supported epithelial production of CX3CL1 (Fig. 3D). Automated quantification using a trainable segmentation algorithm further revealed that mPGES-1 inhibition significantly increased epithelial CX3CL1 expression as compared to VEH-controls (Fig. 3E). To validate mPGES-1-dependent CX3CL1 regulation, we exposed murine epithelial E0771 cells in vitro to medium supplemented with peritoneal lavages obtained from mice at day 6 of the peritonitis model. Medium supplemented with lavage fluid from CIII-treated animals enhanced *Cx3cl1* mRNA expression in epithelial cells, while supplementation with VEH-control lavages left the chemokine expression unaltered relative to the controls (Fig. 3F). Inhibition of mPGES-1 apparently enhanced epithelial expression of CX3CL1, which adds to increase Mϕ numbers in the peritoneal cavity during resolution of zymosan-induced peritonitis.

### CX3CL1 is critical for Mϕ presence in response to mPGES-1 inhibition

**Fig. 4:**
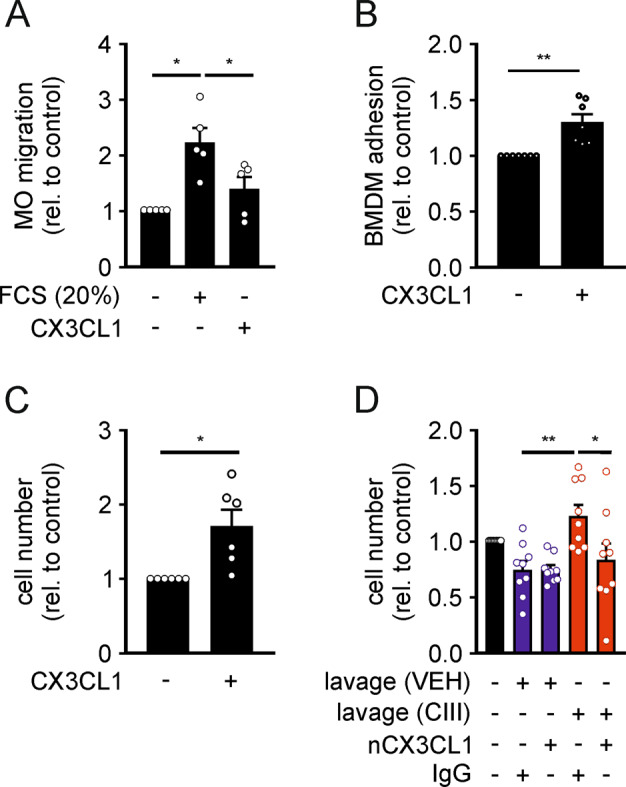
CX3CL1 enhances adhesion and survival of myeloid cells. **A** Murine blood monocytes (MO) were allowed to migrate towards CX3CL1 (100 ng/mL), or medium containing 20% FCS in a Boyden chamber assay for 4 h. The number of MO migrated to the lower compartment was determined by FACS. **B** Bone marrow-derived macrophages (BMDM) were allowed to adhere to fibronectin-coated surfaces in the presence or absence of recombinant CX3CL1 (100 ng/mL). Adhesion was determined after 1 h. **C**, **D** BMDM were grown for 48 h in medium supplemented with **C** recombinant CX3CL1 (100 ng/mL) or **D** peritoneal lavage fluid (1:2) from vehicle control (VEH)- or compound III (CIII)-treated peritonitis mice (day 6) in the presence of either a neutralizing antibody against CX3CL1 (nCX3CL1; 2 µg/mL) or the respective IgG control (IgG) before cell numbers were determined by FACS. Data are presented as means (relative to the respective controls) ± SEM (*n* ≥ 5) and were statistically analyzed using t test or one-way ANOVA with Holm–Sidak posthoc test (*p < 0.05, **p < 0.01).

CX3CL1 is known for its chemoattractive, pro-adhesive, and pro-survival/proliferating effects on mononuclear phagocytes^32–34^. Therefore, we determined the migration-modulating properties of CX3CL1 by allowing MO to migrate for 4 h in a Boyden chamber transwell assay towards CX3CL1 (100 ng/mL). While MO migrated towards the positive control (20% FCS), they did not migrate towards CX3CL1 (Fig. 4A and Supplementary Fig. 2A). Since *Itgb7* ranged high among the DEGs upregulated at day 6 upon CIII-treatment (Fig. 2G), we addressed changes in Mϕ adhesion to fibronectin (FN) in response to CX3CL1. To this end, differentiated bone marrow-derived macrophages (BMDM) were allowed to adhere to FN-coated surfaces. After 1 h we observed significantly more adherent BMDM after stimulation with CX3CL1 (100 ng/mL) compared to controls (Fig. 4B and Supplementary Fig. 2B). To assess if CX3CL1 might also affect Mϕ numbers by altering their proliferation or survival, we stained BMDM with carboxyfluorescein succinimidyl ester (CFSE) and determined both cell number as well as the reduction in mean CFSE (as an indicator of proliferation) after 48 h in the presence or absence of CX3CL1 (100 ng/mL). While proliferation was not affected by CX3CL1 (Supplementary Fig. 2C), higher Mϕ numbers were observed in response to CX3CL1 (Fig. 4C). Furthermore, supplementing media with day 6 peritoneal lavages of zymosan- and CIII-treated mice, resulted in higher cell numbers compared to supplementation with lavages from VEH-treated animals (Fig. 4D), while the proliferation rates remained unaltered (Supplementary Fig. 2D). To test if CX3CL1 within the peritoneal lavages might be involved, we depleted CX3CL1 using a neutralizing antibody (nCX3CL1, 2 µg/mL). This approach completely abolished increased cell numbers in response to lavages of CIII-treated animals (Fig. 4D). Corroborating previous reports that CX3CL1 enhances survival by inducing expression of anti-apoptotic Bcl2 (ref. ^34,35^), *Bcl2* expression was elevated in F4/80^hi^ Mϕ upon mPGES-1 inhibition (Supplementary Table 2). These observations further support the notion that elevated CX3CL1 levels in response to mPGES-1 inhibition contributed to increase Mϕ numbers during late-phase resolution, likely by enhancing their adhesion and survival.

**Fig. 5:**
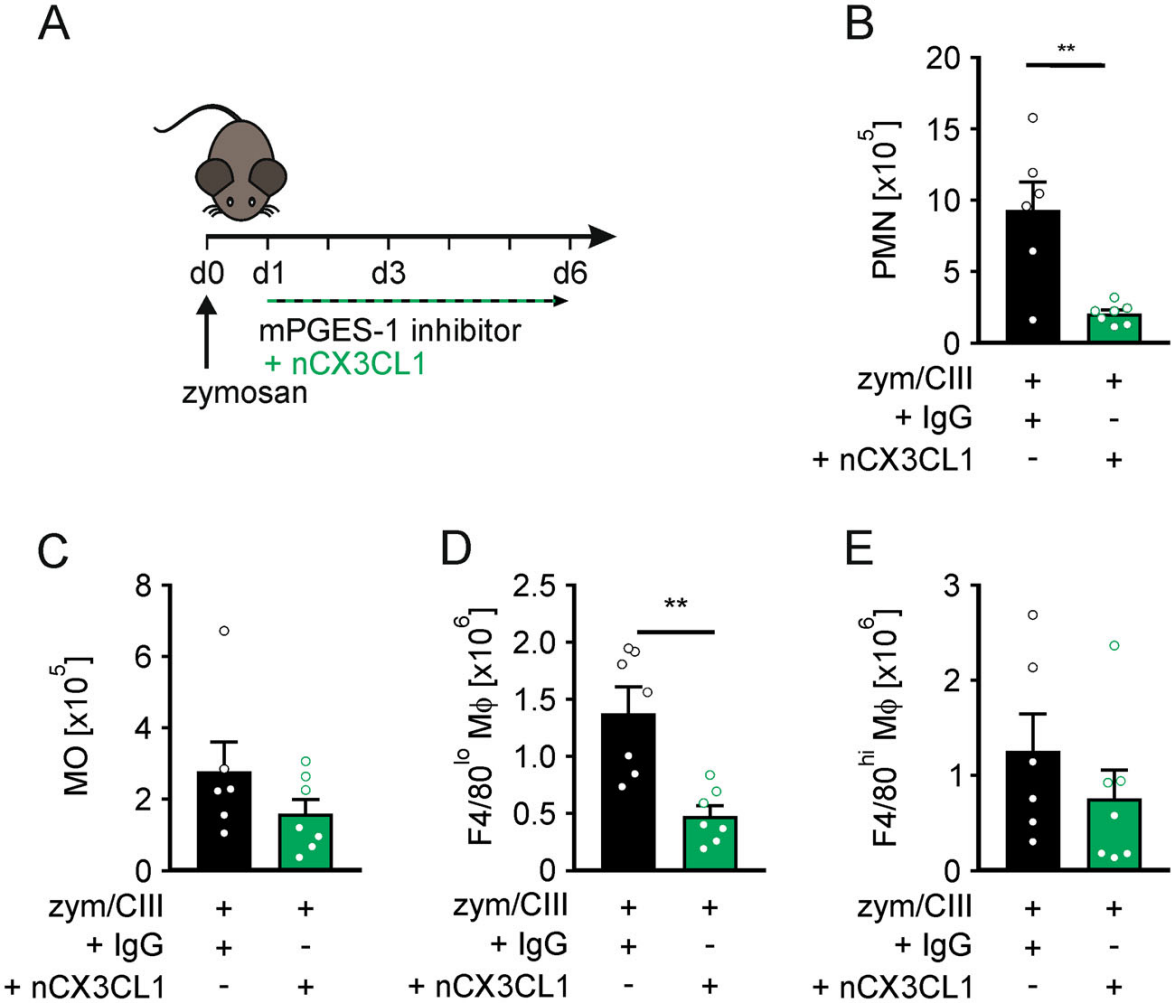
CX3CL1 elevates macrophage numbers during resolution of peritonitis upon mPGES-1 inhibition. **A** Schematic representation of the in vivo CX3CL1-neutralization approach. Starting 24 h post i.p. zymosan (5 mg/kg) injection, mice received daily i.p. injections of the mPGES-1 inhibitor compound III (CIII) (25 mg/kg) in combination with a CX3CL1 neutralizing antibody (nCX3CL1, 5 µg) or the respective IgG control (IgG). Numbers of viable **B** PMNs (CD11b^+^, Ly6G^+^), **C** monocytes (MO; Ly6G^lo^, F4/80^lo^, MerTK^lo^, MHCII^-^), **D** F4/80^lo^ (Ly6G^lo^, F4/80^lo^, MerTK^lo^), and **E** F4/80^hi^ (Ly6G^lo^, F4/80^hi^, MerTK^hi^) macrophages (Mϕ) in the peritoneum at day 6 were analyzed by FACS. Data are presented as means ± SEM (*n* ≥ 6) and were statistically analyzed using t test (**p < 0.01).

To gain evidence that this concept contributes to the situation in vivo, we administered a neutralizing CX3CL1 antibody (nCX3CL1, 5 µg/day) or the appropriate IgG control (IgG) antibody in combination with the mPGES-1 inhibitor CIII in the peritonitis model (Fig. 5A). Neutralizing CX3CL1 significantly reduced PMN numbers in the peritoneum (Fig. 5B), pointing to normalization of the resolution process. Eliminating CX3CL1 from the system left MO numbers unaltered (Fig. 5C), supporting the relevance of enhanced adhesion and/or survival of Mϕ, rather than MO recruitment. Neutralizing CX3CL1 predominantly reduced the number of F4/80^lo^ Mϕ, while F4/80^hi^ Mϕ remained largely unaffected (Fig. 5D, E), despite a marked reduction of CX3CL1 levels (Supplementary Fig. 3).

Conclusively, mPGES-1 contributes to terminate a zymosan-induced peritonitis by limiting epithelial expression of CX3CL1, thereby reconstituting the cellular composition of the peritoneal cavity.

## Discussion

Inflammation is considered a protective mechanism during host defense. To prevent excessive damage of inflamed tissues it is critical to terminate inflammatory processes for efficient recovery^7,36,37^. One of the hallmarks of inflammation and its resolution is the presence and subsequent removal of neutrophils (PMN) at sites of injury. Our finding that the clearance of PMN was attenuated upon mPGES-1 inhibition is in line with a previous report suggesting that PGE_2_ promotes the removal of PMN from the site of injury^18^. In this model, PGE_2_ induced the production of 12-lipoxygenase (12-LO)-dependent pro-resolving lipid mediators in Mϕ, which then contributed to the reverse migration of PMN. While this observation supports the notion that the pro-inflammatory lipid mediator PGE_2_ bears resolution properties^15–20^, we did not observe altered expression of 12/15-LO in response to mPGES-1 inhibition, arguing against this axis in our model. Instead, elevated PMN numbers correlated with the increased presence of MO and Mϕ during late-stage resolution upon mPGES-1 inhibition, suggesting that the latter rather support PMN infiltration and, thus, hinder the reestablishing of homeostatic conditions. Along the same lines, excess Mϕ numbers at sites of inflammation have been shown to favor fibrosis^38,39^, an established outcome of incomplete resolution of acute inflammation. Interestingly, while CX3CR1-expressing Mϕ appeared to promote fibrosis, Mϕ expressing low levels of CX3CR1 rather contributed to wound healing and tissue repair^27,31,40^. Thus, elevated *Cx3cr1* expression in Mϕ late during inflammation upon mPGES-1 inhibition is in line with reports suggesting that PGE_2_ contributed to alternative Mϕ polarization and wound healing^41^. Accordingly, Cox-2 inhibitors reduced the ability of myofibroblasts to induce alternative activation of Mϕ^42^. Since Cox-2 inhibitors attenuate the production of all prostanoids, we feel confident that the use of a mPGES-1 inhibitor allows delineating the role of PGE_2_ more specifically. While mPGES-1 inhibitors were reported to redirect the PGE_2_ precursor PGH_2_ to other prostanoids in vitro^26,43^, we did not observe such shunting in vivo, in line with other reports^44,45^. Epithelial CX3CL1 expression further corroborates previous reports, demonstrating that CX3CL1 is produced by peritoneal epithelial upon peritoneal dialysis, where it contributed to a fibrotic phenotype^27,46^.

With respect to elevated Mϕ numbers upon mPGES-1 inhibition, we observed enhanced adhesion of BMDM in response to CX3CL1, which corroborates previous findings that CX3CL1 increases leukocyte adhesion^47^. Interestingly, *integrin β7* (*Itgb7*) expression was upregulated in both F4/80^lo^ infiltrating and F4/80^hi^ resident Mϕ during zymosan-induced peritonitis upon mPGES-1 inhibition, and *Itgb7* expression was previously associated with adhesion of B-cells^48^ and multiple myeloma cells^49^. Thus, elevated Mϕ numbers upon mPGES-1 inhibition might in part be due to enhanced adhesion of Mϕ within the peritoneum.

During the course of acute inflammation, F4/80^hi^ resident Mϕ are usually depleted at the site of injury by emigration and cell death^5,18^. We noticed that inhibition of mPGES-1 effectively prevented depletion of F4/80^hi^ Mϕ, and, in addition to enhanced *Itgb7* expression, we observed upregulation of anti-apoptotic *Bcl2*, selectively in these cells, which confirms previous reports showing that CX3CL1 enhanced *Bcl2* expression and promoted survival of MO and MO-derived Mϕ^34,50^. The observation that the increase in peritoneal myeloid cells upon mPGES-1 inhibition was reversed upon CX3CL1 neutralization further underscores the crucial role of PGE_2_ to limit CX3CL1 signaling for proper resolution of inflammation. While CX3CL1 neutralization completely reversed F4/80^lo^, infiltrating Mϕ numbers, F4/80^hi^ Mϕ were less responsive. Considering that the neutralization approach lowered peritoneal CX3CL1 levels by approximately 50%, these findings may indicate that the CX3CL1-dependent survival increase of F4/80^hi^ Mϕ is activated at lower CX3CL1 levels, while the adhesion phenotype appears to require higher CX3CL1 concentrations.

In conclusion, we provide evidence that mPGES-1-derived PGE_2_ contributes to the resolution of zymosan-induced peritonitis by reducing the expression of epithelial CX3CL1, which allows to reestablishing cellular homeostasis in the peritoneum.

## Material and methods

### Chemicals

All chemicals were purchased from Thermo Fisher Scientific GmbH (Dreieich, Germany), if not indicated otherwise. Primers were ordered from Biomers (Ulm, Germany).

### Peritonitis model

To analyze the course of inflammation in vivo, we used the well-established zymosan-induced peritonitis model in mice^51,52^. Specifically, to induce a transient peritonitis, 8–12 week old female C57BL/6 mice received an i.p. injection of 5 mg/kg body weight zymosan A (Sigma Aldrich, St. Louis, USA). Starting at day 1 after zymosan injection, mice were randomly selected to receive daily i.p. injections of either the mPGES-1 inhibitor compound III (CIII) (25 mg/kg) or the respective vehicle control (1% Tween-80, 0.5% Carboxymethyl cellulose, 0.9% NaCl solution). To neutralize CX3CL1 a CX3CL1 antibody (5 µg/mouse; MAB571, R&D Systems, Minneapolis, USA) was co-injected with CIII. Peritoneal lavages were obtained by flushing the peritoneal cavity with 3 mL phosphate-buffered saline (PBS). Supernatants and cells of the resulting lavages were used for further analyses. Animal experiments followed the guidelines of the Hessian animal care and use committee (approval number: FU/1211).

### FACS sorting

Single-cell suspensions were stained with an antibody mix (Supplementary Table 3) for 20 min at 4 °C in the dark after blocking with FcR blocking reagent (Miltenyi Biotec, Bergisch Gladbach, Germany) in 0.5% bovine serum albumin (BSA) in PBS as previously described^53^. Cells were separated using a FACS ARIA III cell sorter (BD Biosciences, Heidelberg, Germany) to obtain F4/80^lo^ and F4/80^hi^ Mϕ (gating strategy in Supplementary Fig. 1).

### Cells

Bone marrow-derived Mϕ (BMDM) were differentiated with 20 ng/mL macrophage colony‐stimulating factor (M‐CSF), and 20 ng/mL granulocyte‐macrophage colony‐stimulating factor (GM‐CSF) (both Immunotools, Friesoythe, Germany) in Dulbecco’s Modified Eagle’s Medium (DMEM) (containing 10% fetal calf serum (FCS), 100 U/mL penicillin, 100 µg/mL streptomycin). MO was isolated using the EasySep™ Mouse Monocyte Isolation Kit (Stemcell Technologies, Vancouver, Canada) according to the manufacturer’s instructions. E0771 cells were purchased from ATCC-LGC Standards GmbH (Wesel, Germany), maintained in DMEM containing 10% FCS, 100 U/mL penicillin, and 100 µg/mL streptomycin, and routinely tested for mycoplasma.

### Proliferation assay

BMDM were labeled for 20 min with 5 µM carboxyfluorescein succinimidyl ester (CFSE; Biolegend, San Diego, USA) after 2 days of differentiation. BMDM were then treated with CX3CL1 (100 ng/mL) in the presence of M-CSF or GM-CSF (20 ng/mL each), or exposed to full medium supplemented with lavage fluid isolated at day 6 of the peritonitis experiment at a ratio of 2:1. Cell numbers and CFSE staining were analyzed 48 h post stimulation by FACS.

### Adhesion assay

BMDM were labeled for 20 min with 5 µM CFSE after 7 days of differentiation. BMDM were then allowed to adhere to fibronectin-coated plates in serum-free DMEM (100 U/mL penicillin, 100 µg/mL streptomycin) for 1 h. Adhesion was measured as cell density by quantifying CFSE intensity using the Spark plate-reader system (Tecan, Männedorf, Switzerland).

### Migration assay

Monocyte (MO) migration was assessed in a Boyden chamber transwell assay (5 µm, Corning, USA). Briefly, MO was added to the upper well of a Boyden chamber in DMEM containing 1% FCS, and allowed to migrate towards CX3CL1 (100 ng/mL) in the lower compartment for 4 h. Migrated cells were stained for CD45, CD11b, and Ly6G and analyzed by FACS.

### RNA sequencing

Total RNA was isolated from sorted F4/80^lo^ and F4/80^hi^ macrophages (Mϕ) using the RNeasy Micro Kit (Qiagen, Hilden, Germany) according to the manufacturer’s instructions. RNA concentration and integrity were analyzed with the Qubit HS RNA Assay Kit (Thermo Fisher Scientific) and the Agilent 2100 Bioanalyzer using an RNA 6000 Pico Chip (Agilent Technologies, Waldbronn, Germany), respectively. Sequencing libraries were prepared using the QuantSeq 3′ mRNA-Seq Library Prep Kit with the UMI Second Strand Synthesis Module for QuantSeq (Lexogen, Vienna, Austria). Quantity and quality of the cDNA libraries were evaluated by Qubit dsDNA HS Assay Kit (Thermo Fisher Scientific) and Agilent DNA High Sensitivity DNA Chip (Agilent Technologies), respectively. Libraries were sequenced (single end, 75 cycles) using a High Output Kit v2 on a NextSeq 500 sequencer (Illumina, San Diego, USA). Data were analyzed using the Bluebee QuantSeq FWD-UMI Data Analysis Pipeline according to the manual (details see Supplementary Methods). Sequencing data have been deposited under the GEO accession number GSE164364.

### Reverse transcription and quantitative polymerase chain reaction (qPCR)

Total RNA was isolated from sorted cells using the RNeasy Micro kit (Qiagen), followed by amplification and reverse transcription using the MessageBooster kit (Biozym, Hessisch Oldendorf, Germany). qPCR was performed using the PowerUp SYBR Green Mix on Quantstudio PCR Real-Time Systems (Thermo Fisher Scientific) according to the manufacturer’s manual (primers in Supplementary Table 4).

### Prostanoid analysis

Prostanoids were quantified as previously described^54^ (details are given in Supplementary Methods).

### Immunohistochemistry

Peritoneal membranes from mice at day 6 of the peritonitis model were fixed and paraffin embedded. Deparaffinized and rehydrated membrane sections (4 µm) were stained using the Opal staining system according to the manufacturer’s instructions (Perkin Elmer, Waltham, USA) with primary antibodies against CX3CL1 (MAB571, R&D Systems) and pan-cytokeratin (panCK) (ab7753; Abcam, Cambridge, UK). Pictures were acquired with the Vectra Polaris Automated Quantitative Pathology Imaging System featuring MOTiF (Akoya Biosciences, Marlborough, USA). The relative abundance of CX3CL1 expressing cells in the epithelium was scored upon tissue segmentation based on panCK staining with the InForm software (Akoya Biosciences).

### Statistics

Data are presented as means ± SEM of at least three independent experiments. The sample size for each experiment was estimated empirically, according to the exploratory experiments and published literature with similar methodology. Differences were considered significant when *p < 0.05; **p < 0.01; ***p < 0.001; ns = not significant. Normal distribution was assessed using D’Agostino–Pearson test. Statistical analysis was done using Student’s t test, one-way ANOVA with Holm–Sidak posthoc test (parametric data, otherwise Kruskal–Wallis test), or two-way ANOVA with Holm–Sidak posthoc test (parametric data; otherwise data were log-transformed). Data were statistically evaluated with GraphPad Prism 7.0 (GraphPad Software, USA).

## Supplementary information

Supplementary Information

Suppl. Table 1

Suppl. Table 2

## References

[1] Morgenstern DE, Gifford MA, Li LL, Doerschuk CM, Dinauer MC (1997). Absence of respiratory burst in X-linked chronic granulomatous disease mice leads to abnormalities in both host defense and inflammatory response to Aspergillus fumigatus. J. Exp. Med..

[2] Dinauer MC (1993). The respiratory burst oxidase and the molecular genetics of chronic granulomatous disease. Crit. Rev. Clin. Lab. Sci..

[3] Lauber K, Blumenthal SG, Waibel M, Wesselborg S (2004). Clearance of apoptotic cells: getting rid of the corpses. Mol. Cell.

[4] Dalli J (2008). Annexin 1 mediates the rapid anti-inflammatory effects of neutrophil-derived microparticles. Blood.

[5] Gautier EL, Ivanov S, Lesnik P, Randolph GJ (2013). Local apoptosis mediates clearance of macrophages from resolving inflammation in mice. Blood.

[6] Fadok VA (1998). Macrophages that have ingested apoptotic cells in vitro inhibit proinflammatory cytokine production through autocrine/paracrine mechanisms involving TGF-beta, PGE2, and PAF. J. Clin. Invest.

[7] Stables MJ (2011). Transcriptomic analyses of murine resolution-phase macrophages. Blood.

[8] Dean RA (2008). Macrophage-specific metalloelastase (MMP-12) truncates and inactivates ELR+ CXC chemokines and generates CCL2, -7, -8, and -13 antagonists: potential role of the macrophage in terminating polymorphonuclear leukocyte influx. Blood.

[9] Lantz C, Radmanesh B, Liu E, Thorp EB, Lin J (2020). Single-cell RNA sequencing uncovers heterogenous transcriptional signatures in macrophages during efferocytosis. Sci. Rep..

[10] Watanabe S, Alexander M, Misharin AV, Budinger GRS (2019). The role of macrophages in the resolution of inflammation. J. Clin. Invest.

[11] Serhan CN, Savill J (2005). Resolution of inflammation: the beginning programs the end. Nat. Immunol..

[12] Brown SB, Savill J (1999). Phagocytosis triggers macrophage release of Fas ligand and induces apoptosis of bystander leukocytes. J. Immunol..

[13] Newson J (2014). Resolution of acute inflammation bridges the gap between innate and adaptive immunity. Blood.

[14] Kalinski P (2012). Regulation of immune responses by prostaglandin E2. J. Immunol..

[15] Bystrom J (2008). Resolution-phase macrophages possess a unique inflammatory phenotype that is controlled by cAMP. Blood.

[16] Obermajer N, Muthuswamy R, Lesnock J, Edwards RP, Kalinski P (2011). Positive feedback between PGE2 and COX2 redirects the differentiation of human dendritic cells toward stable myeloid-derived suppressor cells. Blood.

[17] Huang SK, Wettlaufer SH, Chung J, Peters-Golden M (2008). Prostaglandin E2 inhibits specific lung fibroblast functions via selective actions of PKA and Epac-1. Am. J. Respir. Cell Mol. Biol..

[18] Loynes CA (2018). PGE2 production at sites of tissue injury promotes an anti-inflammatory neutrophil phenotype and determines the outcome of inflammation resolution in vivo. Sci. Adv..

[19] Newson J (2017). Inflammatory resolution triggers a prolonged phase of immune suppression through COX-1/mPGES-1-derived prostaglandin E2. Cell Rep..

[20] Tchetina EV, Di Battista JA, Zukor DJ, Antoniou J, Poole AR (2007). Prostaglandin PGE2 at very low concentrations suppresses collagen cleavage in cultured human osteoarthritic articular cartilage: this involves a decrease in expression of proinflammatory genes, collagenases and COL10A1, a gene linked to chondrocyte hypertrophy. Arthritis Res. Ther..

[21] Fuentes AV, Pineda MD, Venkata, Nagulapalli KalyanC (2018). Comprehension of top 200 prescribed drugs in the US as a resource for pharmacy teaching, training and practice. Pharmacy.

[22] Blobaum AL, Marnett LJ (2007). Structural and functional basis of cyclooxygenase inhibition. J. Med. Chem..

[23] Park JY, Pillinger MH, Abramson SB (2006). Prostaglandin E2 synthesis and secretion: the role of PGE2 synthases. Clin. Immunol..

[24] Hara S (2017). Prostaglandin terminal synthases as novel therapeutic targets. Proc. Jpn. Acad. Ser. B Phys. Biol. Sci..

[25] Bergqvist F, Morgenstern R, Jakobsson P-J (2020). A review on mPGES-1 inhibitors: From preclinical studies to clinical applications. Prostaglandins Other Lipid Mediat.

[26] Leclerc P (2013). Characterization of a human and murine mPGES-1 inhibitor and comparison to mPGES-1 genetic deletion in mouse models of inflammation. Prostaglandins Other Lipid Mediat.

[27] Chakarov S (2019). Two distinct interstitial macrophage populations coexist across tissues in specific subtissular niches. Science.

[28] Butenko S (2020). Transcriptomic analysis of monocyte-derived non-phagocytic macrophages favors a role in limiting tissue repair and fibrosis. Front. Immunol..

[29] Combadiere C (1998). Identification of CX3CR1. A chemotactic receptor for the human CX3C chemokine fractalkine and a fusion coreceptor for HIV-1. J. Biol. Chem..

[30] Pan Y (1997). Neurotactin, a membrane-anchored chemokine upregulated in brain inflammation. Nature.

[31] Helmke A (2019). CX3CL1-CX3CR1 interaction mediates macrophage-mesothelial cross talk and promotes peritoneal fibrosis. Kidney Int.

[32] Imai T (1997). Identification and molecular characterization of fractalkine receptor CX3CR1, which mediates both leukocyte migration and adhesion. Cell.

[33] Lucas AD (2003). Smooth muscle cells in human atherosclerotic plaques express the fractalkine receptor CX3CR1 and undergo chemotaxis to the CX3C chemokine fractalkine (CX3CL1). Circulation.

[34] Landsman L (2009). CX3CR1 is required for monocyte homeostasis and atherogenesis by promoting cell survival. Blood.

[35] Chandrasekar B (2003). Fractalkine (CX3CL1) stimulated by nuclear factor kappaB (NF-kappaB)-dependent inflammatory signals induces aortic smooth muscle cell proliferation through an autocrine pathway. Biochem. J..

[36] Weiss SJ (1989). Tissue destruction by neutrophils. N. Engl. J. Med..

[37] Schett G, Neurath MF (2018). Resolution of chronic inflammatory disease: universal and tissue-specific concepts. Nat. Commun..

[38] Wynn TA, Vannella KM (2016). Macrophages in tissue repair, regeneration, and fibrosis. Immunity.

[39] Murray LA (2011). TGF-beta driven lung fibrosis is macrophage dependent and blocked by Serum amyloid P. Int. J. Biochem. Cell Biol..

[40] Burgess M, Wicks K, Gardasevic M, Mace KA (2019). Cx3CR1 expression identifies distinct macrophage populations that contribute differentially to inflammation and repair. Immunohorizons.

[41] Zhang S (2018). Prostaglandin E2 hydrogel improves cutaneous wound healing via M2 macrophages polarization. Theranostics.

[42] Fernando MR, Giembycz MA, McKay DM (2016). Bidirectional crosstalk via IL-6, PGE2 and PGD2 between murine myofibroblasts and alternatively activated macrophages enhances anti-inflammatory phenotype in both cells. Br. J. Pharm..

[43] Xu D (2008). MF63 2-(6-chloro-1H-phenanthro9,10-dimidazol-2-yl)-isophthalonitrile, a selective microsomal prostaglandin E synthase-1 inhibitor, relieves pyresis and pain in preclinical models of inflammation. J. Pharm. Exp. Ther..

[44] Martin EM, Jones SL (2017). Inhibition of microsomal prostaglandin E-synthase-1 (mPGES-1) selectively suppresses PGE2 in an in vitro equine inflammation model. Vet. Immunol. Immunopathol..

[45] Cheng Y (2006). Cyclooxygenases, microsomal prostaglandin E synthase-1, and cardiovascular function. J. Clin. Invest.

[46] Ishida Y (2017). Essential involvement of the CX3CL1-CX3CR1 axis in bleomycin-induced pulmonary fibrosis via regulation of fibrocyte and M2 macrophage migration. Sci. Rep..

[47] Fong AM (1998). Fractalkine and CX3CR1 mediate a novel mechanism of leukocyte capture, firm adhesion, and activation under physiologic flow. J. Exp. Med.

[48] Gorfu G (2010). Beta7 integrin deficiency suppresses B cell homing and attenuates chronic ileitis in SAMP1/YitFc mice. J. Immunol..

[49] Neri P (2011). Integrin β7-mediated regulation of multiple myeloma cell adhesion, migration, and invasion. Blood.

[50] Karlmark KR (2010). The fractalkine receptor CX_3_CR1 protects against liver fibrosis by controlling differentiation and survival of infiltrating hepatic monocytes. Hepatology.

[51] Doherty NS (1985). Intraperitoneal injection of zymosan in mice induces pain, inflammation and the synthesis of peptidoleukotrienes and prostaglandin E2. Prostaglandins.

[52] Cash, J. L., White, G. E. & Greaves, D. R. in *Chemokines, Part B* (Elsevier, 2009), pp. 379–396.

[53] Weichand B (2017). S1PR1 on tumor-associated macrophages promotes lymphangiogenesis and metastasis via NLRP3/IL-1β. J. Exp. Med..

[54] Bärnthaler T (2019). Imatinib stimulates prostaglandin E2 and attenuates cytokine release via EP4 receptor activation. J. Allergy Clin. Immunol..

