# Supplementary material for: Inhibition of mPGES-1 attenuates efficient resolution of acute inflammation by enhancing CX3CL1 expression
Source: Cell Death Dis. 2021 Feb 2;12(2):135. doi: 10.1038/s41419-021-03423-2 (PMC7862376; doi:10.1038/s41419-021-03423-2)
Supplement: Supplementary file 1 — Supplementary Information [file 41419_2021_3423_MOESM1_ESM.docx]

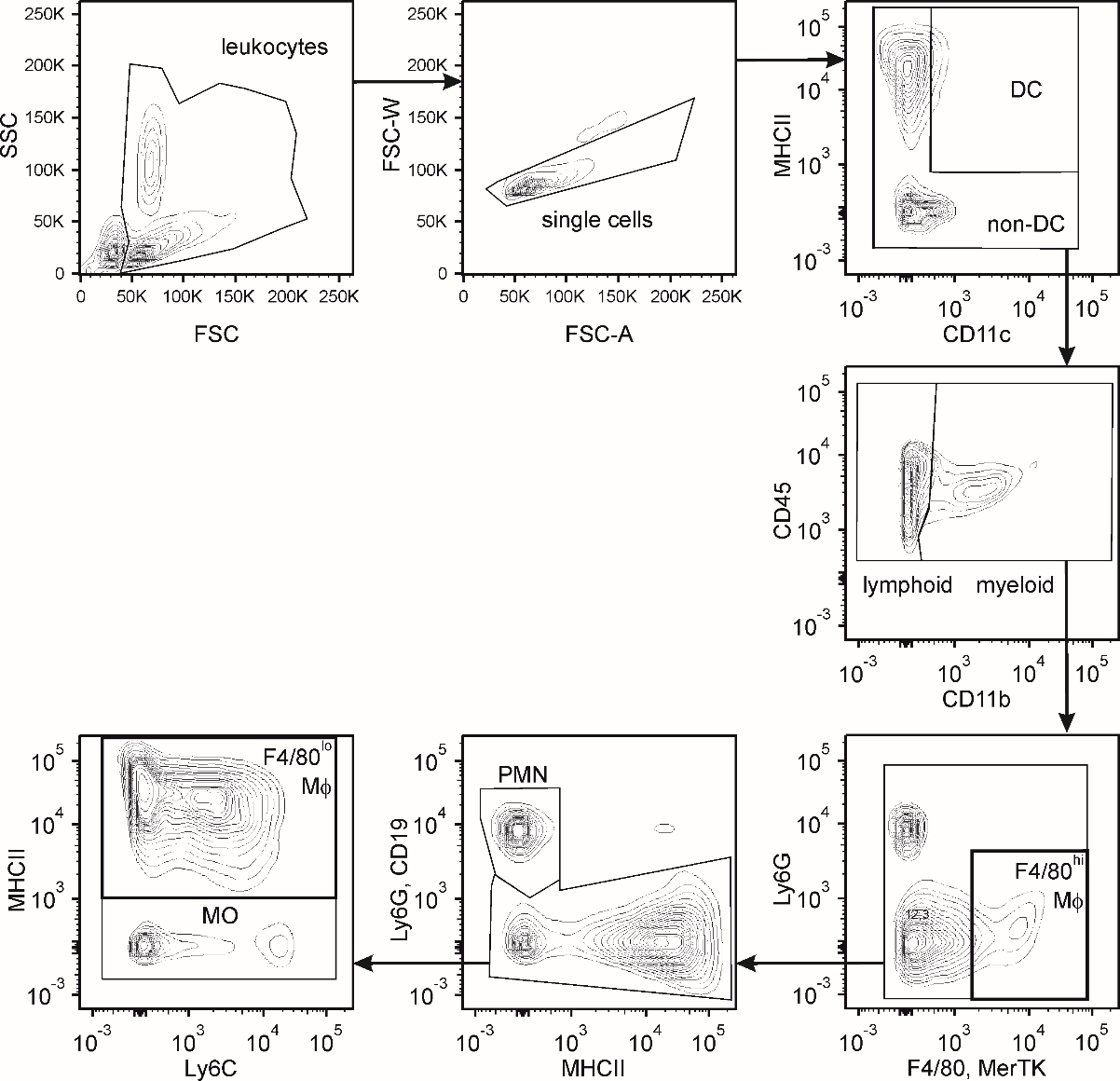
**SUPPLEMENTARY INFORMATION**

**Suppl. Figure 1. Gating strategy of myeloid subpopulations.**

Viable cells and doublets were discriminated by SSC and FSC characteristics. Dendritic cells (DCs) were defined as CD11c^+^, MHCII^+^. CD45^+^ non-DCs were classified as lymphoid (CD45^+^, CD11b^-^) or myeloid (CD45^+^, CD11b^+^) cells. F4/80, MerTK^hi^ cells were defined as F4/80^hi^ macrophages (Mφ) (CD45^+^, CD11b^+^, F4/80, MerTK^hi^). Neutrophils (PMNs) were separated from the remaining cells based on Ly6G expression (CD45^+^, CD11b^+^, F4/80, MerTK^lo^, Ly6G^+^). Within the F4/80, MerTK^lo^, Ly6G^-^ cells, MHCII was used to differentiate F4/80^lo^ Mφ (CD45^+^, CD11b^+^, F4/80, MerTK^lo^, Ly6G^-^, MHCII^+^) from monocytes (MO) (CD45^+^, CD11b^+^, F4/80, MerTK^lo^, Ly6G^-^, MHCII^-^).


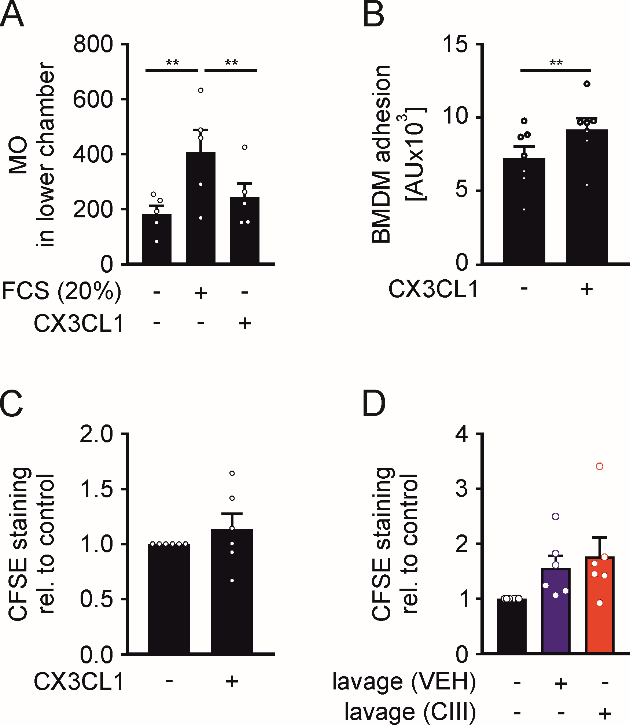

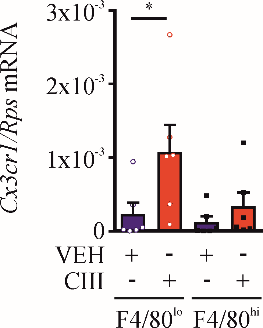


**Suppl. Figure 2. Effect of CX3CL1 on migration, adhesion, and proliferation of myeloid cells.**

**(A)** Murine blood monocytes (MO) were allowed to migrate towards CX3CL1 (100 ng/mL), or medium containing 20% FCS in a Boyden chamber assay for 4 h. The number of MO migrated to the lower compartment was determined by FACS. **(B)** Bone-marrow derived macrophages (BMDM) were stained with carboxyfluorescein succinimidyl ester (CFSE) prior to their adherence to fibronectin-coated surfaces in the presence or absence of recombinant CX3CL1 (100 ng/mL). Adhesion was determined after 1 h by fluorescence measurement after washing. **(C, D)** BMDM were stained with CFSE prior to growing them for 48 h in medium supplemented with **(C)** recombinant CX3CL1 (100 ng/mL) or **(D)** peritoneal lavage fluid (1:2) from vehicle control (VEH)- or compound III (CIII)-treated peritonitis mice (day 6). Proliferation was determined via CFSE intensities by FACS. Data are presented as means ± SEM (n ≥ 5) and were statistically analyzed using t-test or one-way ANOVA with Holm-Sidak posthoc test (**p < 0.01).


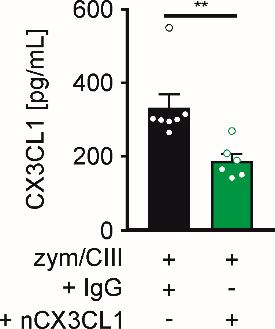


**Suppl. Figure 3. A CX3CL1 neutralizing antibody depletes CX3CL1 in peritonitis.**

Starting 24 h post i.p. zymosan (5 mg/kg) injection, mice received daily i.p. injections of the mPGES-1 inhibitor compound III (CIII) (25 mg/kg) in combination with a CX3CL1 neutralizing antibody (nCX3CL1, 5 µg) or the respective IgG control (IgG). Concentrations of CX3CL1 in the peritoneal lavage were determined by ELISA. Data are presented as means ± SEM (n ≥ 6) and were statistically analyzed using t-test (**p < 0.01).

**Suppl. Table 3: Antibodies used for FACS sorting**

| **Target** | **manufacturer** | **identity** |
| --- | --- | --- |
| Anti-CD11b | Biolegend | Cat# 101257; RRID: AB_2565431 |
| Anti-CD11c | Biolegend | Cat# 117320; RRID: AB_528735 |
| Anti-CD45 | Miltenyi | Cat# 130-102-430; RRID: AB_2751586 |
| Anti-CX3CR1 | Biolegend | Cat# 149025; RRID: AB_2565707 |
| Anti-F4/80 | Biolegend | Cat# 123114; RRID: AB_893478 |
| Anti-HLA-DR (MHCII) | Miltenyi | Cat# 130-102-139; RRID: AB_2660058 |
| Anti-I-A/I-E | Biolegend | Cat# 107614; RRID: AB_313329 |
| Anti-Ly6C | BD Biosciences | Cat# 560525; RRID: AB_1727558 |
| Anti-Ly6C | Biolegend | Cat# 128012; RRID: AB_1659241 |
| Anti-Ly6G | Biolegend | Cat# 127624; RRID: AB_10640819 |
| Anti-MerTK | Invitrogen | Cat# 25-5751; RRID: AB_2573466 |

**Suppl. Table 4: Primers**

| Tata-box binding protein [Tbp] | forward | CTGACCACTGCACCGTTGCCA |
| --- | --- | --- |
|  | reverse | GACTGCAGCAAATCGCTTGGGA |
| Ribosomal protein S6 [RPS] | forward | GAGCGACACTCGGACTTGC |
|  | reverse | GGTATTTCTCGCCATCCACATCT |
| CXC-motif receptor 1 [Cx3cr1] | forward | GAGTATGACGATTCTGCTGAGG |
|  | reverse | CAGACCGAACGTGAAGACGAG |
| CXC-motif ligand 1 [Cx3cl1] | forward | ACGAAATGCGAAATCATGTGC |
|  | reverse | CTGTGTCGTCTCCAGGACAA |

**Supplementary methods**

**RNA seq – bioinformatics analysis.** The Bluebee QuantSeq FWD-UMI Data Analysis Pipeline for Lexogen QuantSeq 3’ sequencing data uses Bbduk from the bbmap suite to trim low quality tails, poly(A) read-through, and adapter contaminations, and identifies and trims UMIs using the umi2index process. Reads were mapped to the Mus musculus reference genome (GRCm38) using the STAR Aligner with modified ENCODE settings followed by UMI collapse to remove PCR duplicates. HTSeq-count is used for gene read counting and differential expression analysis is carried out using the DESeq2 pipeline.

**Prostanoid analysis.** Prostanoid analytics were carried out as previously described (Bärnthaler et al., 2019). Briefly, 200 μL supernatant or plasma was spiked with the isotopically labeled internal standards and extracted using ethyl acetate. The chromatographic separation of the analytes was carried out using a chiral column Lux Amylose-2 (150 x 2 mm inner diameter, 3 μm) coupled to a Synergi Hydro-RP column (150 x 2 mm inner diameter, 4 μm; both from Phenomenex, Aschaffenburg, Germany) under gradient conditions. Water and acetonitrile, both containing 0.1% formic acid, were used as mobile phases and sample run time was 22 min. The MS/MS system consisted of a hybrid triple quadrupole-ion trap mass spectrometer QTrap 5500 (Sciex, Darmstadt, Germany) equipped with a Turbo-V-source operating in the negative electrospray ionisation mode. Analysis was done in the Multiple Reaction Monitoring mode with a dwell time of 50 ms for all analytes. Data were acquired using Analyst Software V 1.6 and quantified with MultiQuant Software V 3.0 (both Sciex), using the internal standard method (isotope dilution mass spectrometry).

**References**

Bärnthaler, T. *et al.* Imatinib stimulates prostaglandin E2 and attenuates cytokine release via EP4 receptor activation. *The Journal of allergy and clinical immunology* **143**, 794-797.e10; 10.1016/j.jaci.2018.09.030 (2019).
